# Assessing equity in health care through the national health insurance schemes of Nigeria and Ghana: a review-based comparative analysis

**DOI:** 10.1186/1475-9276-12-9

**Published:** 2013-01-22

**Authors:** Isaac AO Odeyemi, John Nixon

**Affiliations:** 1 Senior Director and Head of Health Economics & Outcomes Research, Astellas Pharma UK Ltd, Staines, TW18 3AZ, UK; 2 Teaching Associate in Health Economics, Department of Economics and Related Studies, University of York, York, YO10 5DD, UK

**Keywords:** Healthcare systems, Health economics, Health care expenditure, Access, Equity, Social health insurance, National Health Insurance Scheme, NHIS, Sub-Saharan Africa, Nigeria, Ghana

## Abstract

**Background:**

Nigeria and Ghana have recently introduced a National Health Insurance Scheme (NHIS) with the aim of moving towards universal health care using more equitable financing mechanisms. This study compares health and economic indicators, describes the structure of each country’s NHIS within the wider healthcare system, and analyses impacts on equity in financing and access to health care.

**Methods:**

The World Bank and other sources were used to provide comparative health and economic data. Pubmed, Embase and EconLit were searched to locate studies providing descriptions of each NHIS and empirical evidence regarding equity in financing and access to health care. A diagrammatical representation of revenue-raising, pooling, purchasing and provision was produced in order to analyse the two countries’ systems.

**Results:**

Over the period 2000–2010, Ghana maintained a marked advantage in life expectancy, infant mortality, under-5 year mortality, and has a lower burden of major diseases. Health care expenditure is about 5% of GDP in both countries but public expenditure in 2010 was 38% of total expenditure in Nigeria and 60% in Ghana. Financing and access are less equitable in Nigeria as, *inter alia*, private out-of-pocket expenditure has fallen from 80% to 66% of total spending in Ghana since the introduction of its NHIS but has remained at over 90% in Nigeria; NHIS membership in Nigeria and Ghana is approximately 3.5% and 65%, respectively; Nigeria offers a variable benefits package depending on membership category while Ghana has uniform benefits across all beneficiaries. Both countries exhibit improvements in equity but there is a pro-rich and pro-urban bias in membership.

**Conclusions:**

Major health indicators are more favourable in Ghana and overall equity in financing and access are weaker in Nigeria. Nigeria is taking steps to expand NHIS membership and has potential to expand its public spending to achieve greater equity. However, heavy burdens of poverty, disease and remote settings make this a substantial challenge. Ghana’s relative success has to be tempered by the high number of exemptions through taxation and the threat of moral hazard. The results and methods are anticipated to be informative for policy makers and researchers in both countries and other developing countries more widely.

## Introduction

In the countries of Sub-Saharan Africa, health care has evolved along a variety of different lines, which has led to systems today that exhibit a great deal of fragmentation and complexity [1]. In recent years there has been a trend for many developing countries to move towards a new or expanded role for various forms of social health insurance (SHI), including Nigeria and Ghana [2], in the pursuit of universal health care as championed by the World Health Organisation (WHO) [3]. The principal aim is to reduce the high dependency on out-of-pocket(OOP) payments in the form of user charges and co-payments, which are regressive as they disproportionately affect the poorest in society, and therefore challenge the underlying tenets of equity within healthcare systems [1,4].

The evolution of the National Health Insurance Scheme (NHIS) in Nigeria dates back to the post-independence era of 1962 [5]. The government initially funded universal and free health care in predominantly public facilities using revenues from oil exports and general taxation. However, the global slump in oil prices in the 1980s meant that the Government could no longer afford to provide free health care. Several cost recovery mechanisms based on OOP charges were introduced in conjunction with a growth in the privatisation of health care [1]. In addition, the introduction of the Structural Adjustment Programme in 1986 reduced the health sector budget. Additional pressures that led to the introduction of the NHIS included: (1) the general poor state of the nation’s health care services, (2) the excessive dependence and pressure on government-provided health facilities, (3) dwindling funding of health care in the face of rising costs, and (4) poor integration of private health facilities in the nation’s health care delivery system [5]. In terms of implementation [6], the National Council on Health (NCH) approved re-packaging to ensure full private sector participation in the scheme and legislation was signed in May, 1999. The NHIS of Nigeria came into full operation in 2005. Its principal aim is to secure ‘universal coverage and access to adequate and affordable healthcare in order to improve the health status of Nigerians, especially for those participating in the various programmes/products of the Scheme’ [5].

Ghana shares a similar post-colonial history to Nigeria in terms of health care in the country [7,8]. After independence in 1957, the Ghanaian government also introduced a tax-based health financing system in which services were provided free at the point of use by the public sector. By the early 1970s, however, the effects of a stagnating economy meant that the government could not sustain this mode of health financing and delivery. Like Nigeria, the government introduced nominal user fees in the public sector, which in 1985 were raised significantly with the aim of recovering at least 15% of recurrent expenditure. This user fee system, known by the term ‘cash and carry,’ had negative consequences in access to health services, especially for the poorest in Ghanaian society. Limitations included long delays in accessing health services and incomplete prescription purchases. In 2001 the Ghanaian Government passed legislation which established its own NHIS in 2004, with the aim of achieving universal coverage for the population as a whole.

Although these systems have a short history, there is much interest among policy makers regarding their impact on equity and the potential they offer in moving towards universal coverage. The aim of this study is to compare health and economic indicators, describe the structure of each country’s NHIS within the wider healthcare system, and analyse impacts on equity in financing and access to health care. Ghana was selected as a comparator country for Nigeria as evidence suggests that differences may exist in the level of achievement between these two countries [9]. It is anticipated that the results will provide insights to the benefit of both countries and more widely in developing countries.

## Methods

### Literature searches

Searches of Pubmed, Embase and EconLit were undertaken and based on the Boolean expression: [social OR national OR health] AND [insurance OR health care] AND [Nigeria OR Ghana OR Africa OR Sub-Saharan Africa]. The inclusion criteria were that papers should provide either descriptions of the NHIS of the two countries, or provide empirical evidence of equity in financing, access or health care utilisation in the two systems. The selected date range was 2000–2012 due to the fact that NHIS introduction occurred post-2000 in both countries. Searches of the bibliographies of included references revealed further studies that were retrieved. Other relevant references or sources that provided key demographic, health and economic data were also utilised - principally on-line websites of the World Health Organisation (WHO), the World Bank and the official NHIS websites of Nigeria and Ghana. For this analysis a 10-year period (2000–2010) was used to reveal any important trends in the data since the introduction of the NHIS in Nigeria and Ghana. The Organisation for Economic Co-operation and Development (OECD) mean was reported to provide an indication of the potential of each country to develop. The final selection of studies was agreed by the two co-authors.

### Analytical framework

We utilise two well-established definitions of *equity*[2,10], namely (1) *horizontal equity*, which represents the degree to which people who are equals are treated equally; this can apply to *access*, *financial contributions*, *health services utilisation* or *health outcomes* although the latter is at least partly affected by *lifestyle*, *environment* and *human biology* in addition to the healthcare system of a country [11], (2) *vertical equity*, which represents the degree to which people who are different are treated differently.

In terms of equity in financing, the concepts of *progressivity* (whereby payments, as a proportion of income, increase with income) and *regressivity* (when payments, as a share of income, decrease with income and therefore disproportionately affect lower income groups) have been established in seminal studies of OECD countries [10]. Although each country has to be assessed individually, the following general principles can be determined: (1) OOP expenditures are regressive, (2) direct taxation is usually progressive except when tax rates are low, (3) indirect taxes are usually regressive, (4) free market private health insurance is regressive when it plays a dominant or compulsory role, but can be progressive when supplementary to public systems, and (5) statutory SHI is usually (but not always) progressive depending on how contributions are determined.

To represent and describe each NHIS within their respective healthcare systems, two diagrams were developed by the authors using the WHO framework of Murray and Frenk [12]. This framework can be used to present the degree of integration or segmentation (both vertically and horizontally) of the four components of any healthcare system; namely *revenue collection*, *pooling*, *purchasing* and *provision*. This general approach is now widely used in the literature [13,14] but the addition of diagrams facilitates easy assimilation for the reader of how the four functions are either linked or separated from one another.

## Results

The results for all the demographic, economic and health economic indicators over the period 2000–2010 are provided in Table 1. From a total of 147 studies reporting equity issues retrieved by searches, eight met the inclusion criteria for Nigeria and eight for Ghana. The results of these are summarised in the relevant NHIS sections and provided in detail within Tables 2 and 3.

**Table 1:** Key demographic, health and economic indicators - Nigeria, Ghana and OECD mean 2000-2010

	**Demographic Indicators**		**Health Indicators**						**Health Expenditure Indicators**			
**Country**	Population (millions)	GDP (p.cap)	LE (Male)	LE (Female)	IMR	U-5 year MR	p-HIV (% pop)	i-TB (cases)	THE (p. cap)	THE (% GDP)	Public HE (% THE)	OOP HE (% private)
**Nigeria** 2000	**123.7**	**371**	**48**	**47**	**116**	**186**	**3.9**	**172**	**17**	**4.7**	**33**	**93**
Ghana 2000	19.2	260	58	59	64	99	2.3	152	19	7.2	41	80
**Nigeria** 2002	**129.8**	**455**	**47**	**48**	**107**	**177**	**3.8**	**182**	**18**	**4.0**	**26**	**90**
Ghana 2002	20.1	306	58	60	61	94	2.2	138	20	6.5	36	80
**Nigeria** 2004	**136.4**	**644**	**48**	**49**	**102**	**168**	**3.7**	**180**	**44**	**7.0**	**32**	**95**
Ghana 2004	21.1	420	60	61	58	88	2.1	125	26	6.3	35	80
**Nigeria** 2006	**143.3**	**1,014**	**49**	**50**	**97**	**159**	**3.6**	**168**	**59**	**5.7**	**34**	**96**
Ghana 2006	21.1	920	61	63	55	83	1.9	112	48	4.4	57	65
**Nigeria** 2008	**150.7**	**1,374**	**50**	**51**	**93**	**151**	**3.6**	**145**	**80**	**5.7**	**41**	**95**
Ghana 2008	23.3	1,226	62	64	53	79	1.8	99	68	5.6	58	67
**Nigeria** 2010	**158.4**	**1,278**	**51**	**52**	**88**	**143**	**3.6**	**133**	**63**	**5.1**	**38**	**95**
Ghana 2010	24.4	1,325	63	65	50	74	1.8	86	67	5.2	60	66
**OECD 2010**	**N/A**	**34,774**	**77**	**82**	**6.8**	**8.2**	**0.3**	**N/A**	**4,365**	**12.6**	**65**	**67**

**Notes: OECD** = Organisation for Economic Co-operation and Development; **LE** = life expectancy at birth; **IMR** = infant mortality rate per 1,000 live births; **U-5 year MR** is per 1,000 live births; **p-HIV** = prevalence of HIV % of population aged 15–49; **i-TB** = incidence of TB per 100,000; **GDP** = Gross Domestic Product (in 2012 US$); **THE** = total health care expenditure; **p.cap** = per capita; **OOP** = Out of pocket. **Source:** World Bank [15].

**Table 2:** Summary of empirical studies evaluating relevant equity issues for the NHIS (Nigeria)

**Study: Author, date**	**Study Aim**	**Equity Issue/s**	**Sample/s**	**Principal Results**	**Conclusions**
**Adeniyi, 2010**[35]	Assess the knowledge & perceptions of Nigerian dentists to the NHIS	Access to health care	250 dentists employed in private and public dental clinics in Lagos State	61.1% had a fair knowledge of NHIS; 70.4% said NHIS will succeed if properly implemented; 76.6% believed NHIS will improve access to oral health services; 71.4% improve affordability, 68.3% improve availability of services. 74.4% said NHIS oral health care unacceptable.	The majority of the dentists involved in this study had some knowledge of the NHIS and were generally positively disposed towards the scheme and viewed it as a good idea.
**Dienye, 2011**[27]	Determine the pattern of hospital bill payment among rural surgical patients (2005–2009)	Access to health care; financing of health care	229 surgical patients in Ngo; 80% fish farmers & 86% of Christian religion	Multiple sources of finance were used: personal savings (71%), family (49%), organisations (31%), loans (16%), sale of property (30%). Only 3% had knowledge of NHIS, but 84% were willing to enrol.	Sources of finance for payment were multiple but the most common were personal savings & family members. A low knowledge of NHIS contrasted with high willingness to participate.
**Ezeoke, 2012**[4]	Investigate the costs of illness to households in different SES groups & geography; explore payment mechanisms used by different groups	Financing of health care	3,200 households from six communities in two states (Anambra and Enugu in southeast Nigeria)	Malaria was the most common illness. Average cost of transportation for malaria was 86 Naira ($0.6 US), & the total cost of treatment = 2,819.9 Naira ($20 US); drug costs contributed > 90%. OOP payment was the main method of payment. Treatment costs differed by geographic location and SES.	There is the need to substitute OOP spending with pre-payment mechanisms, with cross-subsidies from the rich to the poor & from the healthy to the unhealthy. This can be achieved by expanding NHIS for vulnerable groups, informal sector & scaling up CBHI schemes.
**Ibiwoye, 2008**[9]	Assess the contribution of NHIS to health care delivery; evaluate participation in and use of the NHIS	Access to health care	5,126 employees in the formal sector in Lagos State	11% saw cost as a barrier to membership; 36% had not heard of NHIS; NHIS users were 31.6% in 2006; Concern raised about HMOs & providers; gender, age, income, marital status, family size, education & occupation were significant explanatory variables of NHIS participation.	Low awareness affects NHIS participation and need to promote access, particularly among educated couples. Participation may be improved through compliance of compulsory enrolment and NHIS awareness campaigns.
**Mohamed, 2011**[38]	Determine enrollee satisfaction with provision under the NHIS and the factors influencing satisfaction	Access to health care	280 NHIS university staff enrolees of FSHIP who were insured for more than one year in Zaria-Nigeria	High satisfaction rate with NHIS = 42.1%. Marital status, general knowledge & awareness of contributions positively influenced clients’ satisfaction (p<0.05). Length of employment, salary income, hospital visits and duration of enrolment slightly influenced satisfaction.	The findings have assisted amendment re-prioritization of the operation of the NHIS. Future planning efforts should consider client satisfaction and the factors which influenced it on a regular basis.
**Olugbenga-Bello, 2010**[33]	Determine knowledge & attitude of civil servants in Osun state towards the NHIS	Access to health care; financing of health care	380 civil servants in the employment of Osun state government	40% were aware of NHIS through mainly TV/ billboards. None had good knowledge of the components of NHIS, 26.7% knew about its objectives, 30% knew about who should benefit from the scheme. OOP = 74.7% of health care spending. 0.3% have benefited from NHIS but 52.5% agreed to participate in the NHIS.	A significant association exists between willingness to participate in the NHIS scheme and awareness of methods of options of health care financing and awareness of NHIS.
**Onwujekwe, 2011**[37]	Examine socio-economic & geographic differences in health seeking & expenditures; inform interventions that reduce inequity in utilisation	Access to health care; financing of health care;	4,873 households (2,483 urban and 2,390 rural) in southeast Nigeria	Malaria & hypertension were major diseases requiring OPD and IPD. Providers: PMDs (41.1%), private hospitals (19.7%), pharmacies (16.4%). Rural dwellers & poorer SES groups mostly used low-level & informal providers. Monthly expenditure in urban area = 2444 Naira (US$20.4) & 2267 Naira (US$18.9) in rural area.	Inequities exist in use providers & expenditures on treatment. Reforms should decrease barriers to access public & formal health services & identify constraints which impede the equitable distribution and access for poor & rural dwellers.
**Oyibo, 2011**[34]	Assess the constraints and implications of OOP payments	Financing of health care	247 government employees in Abakaliki, Ebonyi State, south east Nigeria	62.8% reported illness in their in previous 4 weeks; 69% of these used OOP payments, 28.4% used NHIS, 2.6% borrowed money. 63.6% of OOP users had difficulties accessing quality health care; 47.7% used self- medication, 28.4% delayed seeking treatment, 17.1% used herbalists, 6.8% ignored illness.	Most government employees and their dependants in Abakaliki have difficulties in accessing quality health care services with OOP payments. This leads to negative health and access consequences. NHIS enrolees had little difficulty accessing health care.

**Notes: NHIS** = National Health Insurance Scheme; **OPD** = Out-patient department; **IPD** = In-patient department; **PMD** = Patent medicine dealers; **SES** = Socio-economic status.

**Table 3:** Summary of empirical studies evaluating relevant equity issues for the NHIS (Ghana)

**Study Author, date**	**Study Question**	**Equity Issue/s**	**Sample/s**	**Principal Results**	**Conclusions**
**Akazili, 2011**[20]	Analyse the distribution of health care financing in relation to ability to pay	Financing of health care	Ghana Living Standard Survey (GLSS) 2005/2006; Ministry of Finance and other relevant sources; primary household data from six districts	Financing is progressive due to progressivity of taxes (50% of funding). NHI levy is mildly progressive; formal sector NHI payroll deductions are progressive; informal sector NHI contributions are regressive. OOP payments (45% of funding) are regressive.	Extension of pre-payment cover to all in the informal sector is needed - possibly through tax. The pre-payment funding pool for health care needs to grow so budgetary allocation to the health sector can be enhanced.
**Aryeetey, 2012**[40]	Analyse strategies to identify poor for exemptions: means testing (MT), proxy means testing (PMT), participatory wealth ranking (PWR), geographic targeting (GT)	Access to health care	145–147 households per setting: urban, rural and semi-urban in Ghana	Cost of exempting one poor individual = US$15.87 to US$95.44; MT was most efficient and equitable in rural and urban settings with low-poverty incidence; GT was optimal in the semi-urban setting with high-poverty incidence. PMT and PWR were less equitable and inefficient although feasible in some settings.	MT is recommended in low-poverty urban and rural settings and GT is optimal strategy in high-poverty semi-urban setting. The study is relevant to other low-income countries that require identification and exemptions of the poor in social health insurance programmes.
**Jehu-Appiah, 2011**[42]	Identify & compare perceptions of insured & uninsured on NHIS; Explore association with decisions to voluntarily enrol & remain insured	Access to health care	Household survey of 3,301 households and 13,865 individuals	Scheme factors have the strongest association with voluntary enrolment & retention in NHIS (benefits, convenience & price) of NHIS. Negative on price of NHIS, provider attitudes and peer pressure. The uninsured are more negative about these factors.	Perceptions about providers, scheme factors & community attributes are important in household decisions to voluntarily enrol in the NHIS. Policy makers need to design interventions to address these and stimulate enrolment.
**Jehu-Appiah, 2011**[25]	Evaluate equity in enrollment in the NHIS; assess determinants of demand across socio-economic groups	Access to health care	Household survey of 3,301 households	Evaluation included: quality of care, service delivery, provider attitudes, benefits, price & convenience of NHIS, peer pressure & attitudes.’ Results show evidence of inequity as differences exist between the rich and the poor.	Better identification of the poor is needed & premium exemptions should be aggressively pursued. Scheme factors influence decisions to enrol & quality of care should be addressed to retain the rich. SES is a significant factor.
**Mensah, 2010**[44]	Evaluate MDGs 4 & 5 for mothers who are enrolled in the NHIS compared with those who are not	Access to health care	Women (18–49 years) from Brong Ahafo and Upper East. 400 NHIS members &1,600 non-members	NHIS women are more likely to receive prenatal care, deliver at a hospital, have their deliveries attended by trained health professionals, and experience less birth complications.	The NHIS is an effective tool for improving health outcomes among those who are covered. The government should promote further enrolment, in particular among the poor.
**Nguyen, 2011**[8]	Evaluate the impact of the NHIS on households’ OOP spending and catastrophic health expenditure	Access to health care; financing of health care	Household survey in two rural districts, Nkoranza and Offinso	NHIS coverage (2007) was 35%; OOP payment for care from informal sources & for uncovered drugs and tests occurred in NHIS but significantly less than the uninsured. Effect was strong among the poorest in the sample.	NHIS gives a positive financial protection effect, stronger among the poor. Social health insurance cannot fully remove OOP payments. Further work is needed on supply-side incentives & quality of care.
**Sarpong, 2010**[43]	Explore the association between socio-economic status (SES) and NHIS membership	Access to health care	Residents of the Asante Akim, north district of the Ashanti region (99 villages, 7,223 households)	38% subscribed to the NHIS, of these 21% were low, 43% middle and 60% high SES households. SES was significantly associated with NHIS subscription (high SES: Odds Ratio [OR] = 4.9 low SES OR = 1, reference group).	To achieve universal access to health care facilities for all residents of Ghana, in particular for individuals living under socio-economic constraints, increasing their subscription rates is necessary.
**Witter, 2009**[30]	Assess the NHIS (2005 to 2009) to inform NHIS developments & other innovations in the region	Financing of health care; access to health care	Literature plus stakeholder interviews at national, regional and district levels	NHIS is reliant on tax (70–75%); large exempted population (30%) ; coverage rose from 7% to 45%; growth in distressed schemes; VAT-based source is regressive; membership of NHIS is pro-rich & pro-urban; ‘squeezing out’ of non-members from health care utilisation; strengthening of purchasing needed.	Some trade-offs will be necessary to achieve universal coverage. In the long term, investment in the NHIS will only be justified if it is able to increase the cost-effectiveness of purchasing and the responsiveness of the system as a whole.

**Notes: NHIS** = National Health Insurance Scheme; **OPD** = Out-patient department; **IPD** = In-patient department; **PMD** = Patent medicine dealers; **SES** = Socio-economic status; **OOP** = out-of-pocket; **MDG** = Millenium Development Goal; **VAT** = Value-added Tax; **OR** = Odds Ratio.

### Demographics

Nigeria is a Lower Middle-Income Country (LMIC) in West Africa [15] with a population that has risen from 123.7 million in 2000 to158.4 million in 2010. 48.1% of the population live in rural areas and 51.9% in urban areas [16]. Ghana is also a LMIC country [15] in West Africa but has a much smaller population that rose from 19.2 million in 2000 to 24.4 million in 2010. Like Nigeria it has 50% of its population located in rural areas.

Table 1 indicates strong economic growth with GDP per capita (US$) rising from $371 in 2000 to $1,278 in 2010 for Nigeria. The annual growth rate for 2010 was an impressive 8.7%. Similar levels of growth are observed for Ghana, rising from $260 in 2000 to $1,325 in 2010. The annual growth rate for 2010 was also impressive at 7.7%. The OECD mean was $34,774 in 2010, which reflects the potential for further growth in the economies of Nigeria and Ghana.

### Health indicators

The six health indicators in Table 1 provide a useful overview of the two countries as they are commonly used in international comparisons [17]. Three variables form part of the eight health-related United Nations’ Millennium Development Goals (MDGs) [18].

In 2000 life expectancy in Nigeria was 48 years for males and 49 years for females. The corresponding figures for Ghana were 58 and 59 years, respectively. Both countries experienced year-on-year rises and in 2010 life expectancy for males in Nigeria increased to 51 years compared with 63 years in Ghana, and female life expectancy had risen to 52 years in Nigeria and 65 years in Ghana. The greater advantage for Ghana has therefore been maintained or expanded over the period of analysis. For comparison, the OECD mean in 2010 was 77 years for males and 82 for females.

Infant mortality rate and under-5 year child mortality rate are closely related and linked to the 4^th^ MDG, which is to *reduce the under-5 child mortality rate of each country by two thirds between 1990 and 2015*[18]. In Nigeria for 2000, infant mortality rate per 1,000 live births was 116 and under-5 mortality was 186. Ghana shows a substantial advantage with figures of 64 and 99, respectively. Over the period 2000–2010, Nigeria and Ghana experienced year-on-year improvements such that in 2010 infant mortality rate and under-5 mortality rate fell to 88 and 143 for Nigeria, respectively, and for Ghana the figures were 50 and 74, respectively. Using World Bank health data [15] the target MDG for 2015 can be calculated to be 70 deaths per 1,000 live births for Nigeria as it was 213 in 1990, which means considerable progress is needed in order to meet this goal [18]. Using the same calculation, Ghana is making good progress to meet its target of 40 in 2015, from 122 in 1990, but again this will be a major challenge to achieve [19]. The OECD mean for these two variables in 2010 was 6.8 and 8.2 per 1,000 live births, respectively.

In terms of major disease burden indicators, Table 1 reports the prevalence of HIV as a percentage of the population aged 15–49, and the incidence of tuberculosis (TB). These variables are closely linked with MDG goal 6 which is to *combat HIV/AIDS, malaria and other diseases*. In Nigeria, HIV prevalence fell from 3.9% to 3.6%, and the incidence of TB fell from 172 to 133 cases per 100,000. For Ghana, HIV prevalence fell from a lower base of 2.3% to 1.8%, and the incidence of TB fell, again from a lower base of 152 cases to 86 cases per 100,000 in 2010. Data from the World Bank indicate that, in 2008, the number of new cases of malaria per 100,000 of population was 38,259 for Nigeria and 31,179 for Ghana.

The general pattern, therefore, is that Nigeria has a higher disease burden but a good deal of progress is being made for both countries.

### Health expenditure indicators

The health expenditure indicators in Table 1 are chosen to reflect three aspects of funding: (1) the level of commitment governments have towards the funding of health care, (2) the level achieved per capita, which reflects the economic strength of a country in relation to the size of its population, (3) public health expenditure as a share of total health expenditure, which reflects the level of government financing of health care, and (4) the percentage of private health expenditure provided by OOP contributions, which reflects the contributions made by individuals.

Total health expenditure as a percentage of GDP increased from 4.7% in 2000 to 5.1% in 2010 for Nigeria, but an inconsistent trend is observed in that it reached 7% in 2004 but had fallen to 5.7% in 2006 and 2008. In contrast, Ghana was spending 7.2% on health in 2000 but in 2010 this had fallen to a similar figure to Nigeria at 5.2%. Compared with the OECD mean of 12.6%, the level for both Nigeria and Ghana is less than half that of developed countries. The data for per capita total health care expenditure indicate that in 2000 Nigeria spent US$ 17 and Ghana US$ 19, which generally rose year-on-year in both countries to reach US$ 63 in Nigeria and US$ 67 in Ghana.

Data for public health expenditure as a percentage of total health expenditure indicate a large difference. In Nigeria the figure rose slightly from 33% in 2000 to 38% in 2010, while in Ghana the respective figures were 41% and 60%. This reveals a much stronger role for the government in Ghana in terms of funding health care and much closer to the 65% achieved in OECD countries. It should be noted that since the introduction of the NHIS in Ghana (2004) public expenditure has risen quite sharply from 35% to 60% while in Nigeria a more modest increase is observed from 32% to 38% over the same period.

The final column of Table 1 reports the percentage of private expenditure that comes from OOP sources in the form of user charges and/or co-payments for treatment. In Nigeria for 2000 this was very high at 93% but even higher (95%) in 2010. In contrast, OOP expenditure was 80% in 2000 for Ghana until the introduction of the NHIS in 2004, and fell sharply to 66% by 2010. The OECD mean is 67%, which is almost identical to the figure for Ghana.

These findings suggest that the financing of health care in Ghana has become less regressive since the introduction of the NHIS, whereas in Nigeria very high OOP payments as a share of private health expenditure have persisted.

#### The NHIS of Nigeria and Ghana within their respective healthcare systems

**Figure 1:**
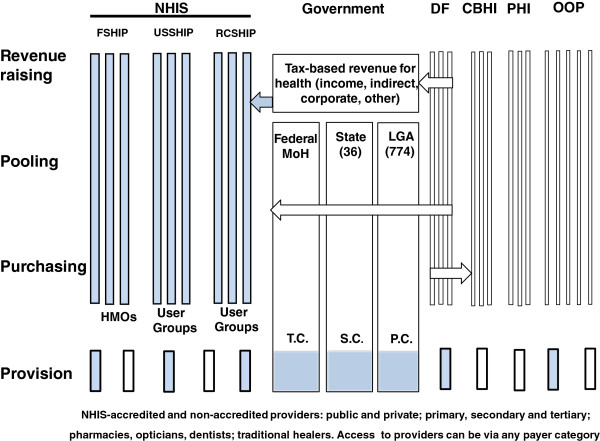
**The NHIS of Nigeria: revenue raising, pooling, purchasing and provision. Source:** Developed by the authors based on Murray and Frenk [12] and studies/references from the review. **Notes:** The relative size of each element does not equate to population size; Shaded boxes **=** NHIS elements; **NHIS** = National Health Insurance Scheme; **FSHIP** = Formal Sector Social Health Insurance programme; **USSHIP** = Urban Self-Employed Social Health Insurance Programme; **RCSHIP** = Rural Community Social Health Insurance Programme; **DF** = Donor Funding; **CBHI** = Community-Based Health Insurance; **PHI** = Private Health Insurance; **OOP** = Out-of-pocket, **T.C.** = Tertiary care; **S.C.** = Secondary care; **P.C.** = Primary care; **LGA** = Local Government Authority; **HMO** = Health Maintenance organisation; **MoH** = Ministry of Health

**Figure 2:**
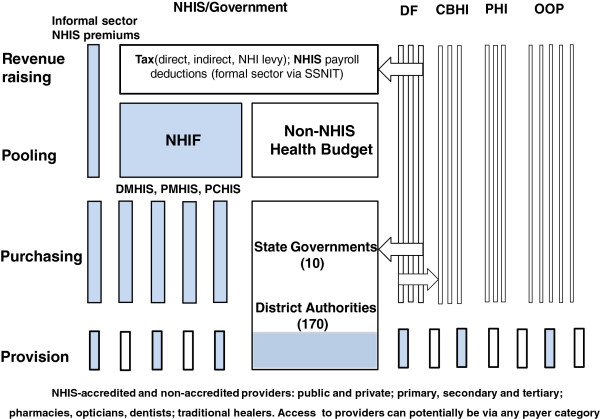
**The NHIS of Ghana: revenue raising, pooling, purchasing and provision. Source:** Developed by the authors based on Murray and Frenk [12] and studies/references from the review. **Notes:** The relative size of each element does not equate to population size; **Shaded boxes =** NHIS elements; **NHIS =** National Health Insurance Scheme; **SSNIT** = Social Security and National Insurance Trust; **DMHIS =** District Mutual Health Insurance Schemes; **PMHIS** = Private Mutual Health Insurance Schemes; **PCHIS** = Private Commercial Health Insurance Schemes

Figure 1 provides an overview of the Nigerian healthcare system while Figure 2 illustrates the system for Ghana. The shaded areas highlight the principal NHIS elements.

As can be seen, there are six elements that contribute to the four healthcare system functions of *revenue raising*, *pooling*, *purchasing* and *provision,* which are described in the following sections. Providers include primary care providers, hospitals (secondary care, tertiary care), pharmacists, chemical shops, maternity clinics, opticians, dentists, diagnostic centres, laboratories and traditional healers (which are common in both Nigeria and Ghana). Providers shown as shaded rectangles are accredited by the NHIS. For simplicity, government-funded provision is shaded as providers are either part of the NHIS (as in tertiary care hospitals, for example) or potentially part of the future NHIS. As the first five elements are common to both countries, they will be described before the NHIS elements.

### Out of pocket (OOP) payments

OOP expenditure is represented by the block of vertical tubes on the right-hand side of Figures 1 and 2. These are horizontally segmented from each other and vertically integrated to the point of purchasing, and depict households or individuals. Pooling can usually only occur among family members and so has limited effect on risk sharing. In Nigeria, the level of OOP expenditure as a share of total health expenditure is very high at around 65% [5]. The figure for Ghana is somewhat lower at about 45% [1].

Evidence from an empirical analysis of OOP payments in Ghana, but generalizable to other similar countries, confirms their regressive characteristic [20]. As such vertical and horizontal equity in financing are more adversely affected by OOP payments in Nigeria than Ghana.

### Private Health Insurance (PHI)

PHI here refers to that provided by free market insurance companies who are not within the NHIS. As shown in Figures 1 and 2, a number of vertically integrated and horizontally segmented companies compete for customers. In the case of Nigeria, some private insurance companies are fully integrated from revenue-raising to provision as they manage their own hospitals or clinics, which appears not be as prevalent in Ghana [21]. The diagrams, however, indicate the most common arrangement of a purchaser-provider split. PHI companies do not pool their revenues with each other to equalise risk. PHI is utilised by a small percentage of the population who may use it to seek faster access to health care, more choice of provider and higher quality facilities. In 2003, before implementation of the NHIS, only 6.7 % of private health expenditure was attributed to private pre-paid insurance plans in Nigeria and current data suggest this amounts to less than 1% (one million people) [22]. In Ghana it appears to have remained at under 1% of the population pre-and post-NHIS implementation [1,23]. Free market PHI often adversely affects equity because of the regressive nature of actuarially fair premiums (set according to risk) and flat rate co-payments and deductibles. Evidence from five African countries, including Ghana and Nigeria, suggests that the uptake of voluntary PHI is by predominantly wealthier individuals from urban communities [21].

### Community-based Health Insurance (CBHI)

CBHI has been a common feature of health care financing in Nigeria and Ghana, especially within the poorer rural communities [24]. Also known as Mutual Health Associations (MHAs), CBHI is not-for-profit health insurance with members regularly paying small premiums into a collective pool of funds, which are then used to pay for health services that members require. Although CBHI may involve hundreds of programmes it only accounts for a very small portion of total health expenditure - for example in Ghana it was only 1% in the early 2000s [1] although the actual number of CBHI schemes grew from 2 in 1995 to 78 in 2004 [21]. Whilst legally mandated to operate, CBHI programmes in Ghana that are outside the NHIS do not receive funding for exempted citizens [25], which may act as a driver for providers to seek accreditation by NHIS programmes as a way of scaling up health insurance coverage.

In terms of equity in financing, CHBI offers limited opportunities to raise sufficient funds for pooling and risk sharing due to the often low enrolment of target populations; one reported study of West African countries showed this varied from 8-82% [24]. Flat-rate payments are mostly used and are therefore regressive. In terms of the package of benefits offered, this can vary widely and may be limited to out-patient care at primary health care facilities [1]. Some findings also suggest that policies intended to promote equity can lead to a reduction in quality of services and that adverse selection (of higher risk participants) is often a feature due to the voluntary nature of CBHI [24].

### Donor Funding (DF)

DF, as shown in Figures 1 and 2, is an important form of revenue-raising for many developing countries such as Nigeria and Ghana, and comes in the form of grants and loans from various external sources [3]. These include contributions from countries with bilateral agreements, multilateral institutions such as the World Bank or the WHO, global health initiatives, and charities or philanthropic organizations. In Figures 1 and 2, DF is shown entering the system either at various government levels to add to the public pool of funds for health care, or passed directly to community programmes at the level of purchasing. In some cases, however, organisations make use of their own resources on the ground as an additional source of provision. While this is often targeted at specific programmes and agencies such as non-governmental organisations (NGOs), in terms of supporting health care as a share of total spending it constituted 4.9% in Nigeria and 14% in Ghana for 2009 [15].

The potential effect of DF is to increase both horizontal and vertical equity in financing and access to health care facilities, particularly for those in poor rural areas.

### Government

The government clearly plays a significant role in the health care sector by raising both direct and indirect taxes and allocating part of it to fund health care.

In the case of Nigeria, it has judicial, legislative and executive arms of government at Federal and 36 State levels. There are 774 LGAs and 9,572 political wards. The three arms of government in Nigeria (Figure 1) have a degree of horizontal and vertical segmentation between them [26]. The Federal Government collects taxes and allocates the health budget to the Ministry of Health (MoH). Allocations are then made to Federal government for the purchasing and provision of public tertiary care across the country with some overlap regarding primary care programmes. State governments also receive and pool funds to purchase secondary care public facilities, while LGAs receive and pool funds to purchase public facilities in the primary care sector. Some funding is shown being passed to the NHIS programme (shaded arrow) to cover exempted groups such as children under 5, the permanently disabled and prison inmates [5]. Collectively, the providers created by the three arms of government contribute to the stock of providers that citizens can access either through OOP arrangements, CBHI, PHI or the NHIS. Vertical integration down to provision is shown in Figure 1 as all arms of government mostly run their own facilities and, for simplicity, no purchaser-provider split is shown.

A recurring criticism relates to the small amount allocated to the health budget in Nigeria - in 2003, for example, it was only 3.2%, which has implications for equity within the system regarding the quantity and quality of health care resources [6,27]. The Abuja summit of 2001 called for African governments to commit 15% of annual government budgets to their health sectors [28]. In the case of Nigeria, therefore, this target remains to be met.

Figure 2 shows that Ghana is divided into ten administrative/political regions, which are further divided into 170 District Authorities (DAs). The DAs develop, plan and mobilize resources for programmes and strategies for the development of the district [29]. Tax contributes about 70% of the funding envelope for the NHIS of Ghana [30] and as such is more strongly integrated with the NHIS compared with Nigeria. The Government has also met the Abuja target of allocating 15% of its budget to health care, which includes NHIS funding [30].

As shown in Figure 2, , pooled taxation funds are allocated to State governments and then DAs, which purchase public health care facilities in primary, secondary and tertiary care and can be accessed, as in the case of Nigeria, via the same categories of provider shown in Figure 2. As in the case of Nigeria and for simplicity, it is assumed that vertical integration exists down to provision in the non-NHIS sector but some contracting arrangements may involve a purchaser-provider split.

#### NHIS (Nigeria)

The role of the NHIS is to regulate, monitor, enforce quality controls and administer the system, including care to the disadvantaged sectors in Nigerian society [5,31]. As can be seen in Figure 1, the NHIS (shaded boxes) is built around three main sub-schemes to cater for different segments of the population:

•The Formal Sector Social Health Insurance Programme (FSHIP);

•The Urban Self-Employed Social Health Insurance Programme (USSHIP);

•The Rural Community Social Health Insurance Programme (RCSHIP)

The **FSHIP** was the first scheme to be rolled out in 2005 and covers the public sector, organised private sector (employers with more than10 employees), the armed forces, Police and allied services, students of tertiary institutions, and voluntary contributors. The FSHIP is implemented by Health Maintenance Organisations (HMOs) and NHIS-accredited providers. HMOs are vertically integrated in revenue-raising, pooling and purchasing, but horizontally segmented from each other. A purchaser-provider split therefore generally exists but some HMOs also run hospitals or clinics. HMOs can either be for-profit or not-for profit private health insurance companies, or public entities. They operate within a competitive framework under the model of ‘managed care,’ which has its roots in the United States as a potentially more cost-effective way of delivering health care in comparison with free market PHI [11,31].

In terms of operation [5], employers affiliate with an NHIS-approved HMO which provides employees (under the age of 60) with a list of NHIS-approved Health Care Providers (HCPs). The employee registers all family members with a HCP of his/her choice and also has the right to change his/her HCP after a minimum period of three months if he/she is not satisfied with the services being given. Contributors and associated dependents are given an identity card with which to obtain health care after a specified waiting period.

Revenue-raising in the FSHIP is shared by the employer and employee, who pay 10% and 5% of the employee’s basic salary, respectively [5]. Contributors may be asked to make a small co-payment, where applicable, at the point of service. Revenue-raising in the FSHIP is therefore reasonably equitable but is likely to be regressive overall because of *proportional* contributions in respect of income with flat rate co-payments and user charges.

Fund pooling, as shown in Figure 1, exists within individual HMOs in order to share risk among its members and make necessary payments. As membership of HMOs varies quite considerably, however, from less than a thousand to tens of thousands [31], the potential to benefit from pooling is somewhat variable. The NHIS in Nigeria has not implemented ‘risk equalisation’ among HMOs – a process that involves financial transfers from insurance companies with a low-risk membership to those with older or sicker members as practiced in controlled market insurance schemes. An example of such mechanisms in developing countries is that of South Africa [13,14].

In terms of purchasing, the HMO makes payments to the HCP for services rendered using one of five alternatives: (1) *capitation*, which involves regular payments in advance of treatment based on membership size, (2) *fee-for-service*, which is mostly applicable to private providers based on ‘authorised referrals,’ (3) *per diem,* namely daily fees for medical treatment during hospitalisation, or (4) *per case* payment. The latter is based on the Diagnostic Related Group (DRG) mechanism [11], which determines the average cost of defined episodes of care in hospital before the patient receives treatment. This facilitates prospective costing for the HMO, and the provider may either gain or lose income depending on how efficiently the patient is treated.

In Figure 1, provision of benefits in the FSHIP covers the employee, a spouse and four biological children below the age of 18 years. As such, polygamous households need to make additional payments. Additional children can be added for additional cost but those over 18 in full time education are covered under the students’ scheme.

Gate-keeping is a feature of the NHIS as the HCP is the first point of contact before referrals are made to secondary or tertiary care. The benefits package covers: *out-patient* care & consumables, *prescribed drugs and diagnostic tests* covered by the National Essential Drugs List and Diagnostic Test Lists, *antenatal and maternity* care for up to four live births, *preventive care* including immunization, *consultation*s with specialists, *hospital care* in a standard ward, *eye examination and care* excluding the provision of spectacles and contact lenses, *prostheses* (artificial limbs), *dental care* and pain relief [5]. Treatment excluded from coverage includes antiretroviral drugs (but these are covered by the National Action Committee for AIDS (NACA)), care for terminal illnesses such as cancer and AIDS, and chronic health problems such as diabetes, renal dialysis and hypertension [5,6].

The **USSHIP** element of the NHIS is a non-profit health insurance programme covering occupation-based User Groups (UGs) with common economic activities and administered by a Board of Trustees. UGs must contain at least 500 members to ensure adequate pooling of financial resources. These are shown in Figure 1 as vertically integrated from revenue-raising to purchasing and horizontally segmented. In relation to revenue-raising, participants pay a flat monthly rate with contributions depending on the health package chosen by members of the UG, which is based on their health needs. As with HMOs, pooling takes place within funds but not between them and therefore membership influences the degree of risk sharing that is possible. Health care benefits are delivered by accredited providers, as in the case of the formal sector.

The **RCSHIP** is also a non-profit health insurance programme for a cohesive group of households or individuals (including retirees) that form a community. The scheme is run by its members using the same management structure as the USSHIP element with vertically integrated, horizontally segmented UGs as shown in Figure 1. Based on the model of Health Mutual Associations (HMAs), the scheme can encompass Community-Based Organisations (CBOs), Faith-Based Organisations (FBOs), Non-Governmental Organisations (NGOs) and Civil-Society Organisations (CSOs). Members of the specified community, based on their health needs, acquire NHIS accreditation and then choose the health care benefits with contributions being made in cash, paid as a flat monthly rate or by means of instalments. This contribution rate will depend on the health package chosen by members of the UG.

In terms of equity, both the USSHIP and RCSHIP offer promising ways of scaling up NHIS participation outside the compulsory formal sector scheme. However, it should be noted that both are voluntary schemes based on variable financing arrangements and benefits packages and require a high degree of self-management and administration. The benefits packages are not fully comprehensive, which means some members will not be able to access care for excluded conditions. The main impact, therefore, is to increase horizontal equity for some participants in financing and provision, but reduce vertical equity for others.

#### NHIS implementation and factors affecting equity

Partly because of the phased implementation of the NHIS in Nigeria, enrolment appears to be rather sluggish as in 2011, there were only 5.3 million Nigerians (about 3.5% of the population) enrolled and the principal participants were those in the FSHIP element, including 600,000 pregnant women and children under the Maternal and Child Health (MCH) project [32]. Although accurate membership data for each of the three programmes are difficult to confirm, total participation, consistent with the above data, is estimated to be at approximately 5 million [22]. A steady increase in HMOs has been observed and there are now 62 that have been accredited and registered, with more applications being processed. In 2012 there were 5,949 accredited HCPs and over 4 million identity cards had been issued [5]. The system is therefore making variable progress but some recent initiatives regarding external sources and new legislation are anticipated to move the situation forward and are addressed in the discussion. In the following sections, evidence from the eight included studies in Table 2 are summarised in conjunction with each aspect of equity that was addressed by them.

### Equity in financing

Four studies assessed equity in financing concerning the consequences of a reliance on OOP payments. Multiple sources of finance were shown to be utilised by surgical patients in Ngo [27] in that respondents had used: personal savings (71%), contributions from family members (49%), organisations (31%), loans (16%) and sale of property (30%). OOP spending among a sample of civil servants in Osum State also showed that it was as high as 74% [33]. Another study of rural dwellers in Anambra and Enugu states [4] found that malaria was the most common illness facing households, with strong evidence that the effect of OOP expenditures falls disproportionately on poorer socio-economic groups and varies according to geographical location.

The negative consequences for equity because of high OOP payments can be seen from a study of government employees in Abakaliki, Ebonyi State, which found that 63.6% of OOP users had difficulties obtaining quality health care; 47.7% used self-medication, 28.4% delayed seeking treatment, 17.1% used herbalists, and 6.8% ignored their illness [34].

### Equity in access

Five studies reported empirical findings regarding access to health care*.* A survey of dentists in Lagos State [35] found that 61% had only a fair knowledge of the NHIS but 76.6% believed it would expand access to dental care by improving affordability and availability of services. However, 74% had concerns about the quality of NHIS treatment currently available.

In a study of rural surgical patients by Dienye *et al.*[27], only 3% of respondents had knowledge of the NHIS but 84% were willing to enrol. In another survey of civil servants in Osun State by Olugbenga-Bello and Adebempi [33] the authors found low levels of knowledge about the NHIS and only 0.3% had actually benefited from it. However, 52.5% said they would participate in it.

Another study by Oyibo [34], which addressed the issue of access constraints for government employees in Abakaliki, Ebonyi State, found that NHIS enrolees had little difficulty in accessing health care compared with those relying on OOP payments. One large-scale study of formal sector employees in Lagos [9] found that only11% saw cost as a barrier to membership, but 36% had not heard of the NHIS even though membership among them rose from 4.5% in 2000 to 31.6% in 2006. Gender, age, income, marital status, family size, education and occupation were significant explanatory variables regarding access to health care through NHIS participation. The authors made the point that participation could be improved through compliance with compulsory enrolment for the formal sector, and indicated that the informal sector requires an NHIS awareness campaign as membership is not mandatory.

In terms of access being hindered by payment mechanisms*,* while capitation payments were found to be reliable, some studies reported delays in providers receiving authorization to offer services to clients using fee-for-service payments. Providers had also experienced delays in receiving approval to refer patients across levels of care [36].

Concern was raised about the service provided by HMOs and providers by a sample of employees in the formal sector [9]. A study by Onwujekwe and Onoka [37] also revealed that rural dwellers and poorer socio-economic groups mostly accessed low-level and informal providers.

Evidence on the satisfaction levels for NHIS participants was reported in a study by Mohamed *et al.*[38], which surveyed FSHIP enrolees in Zaria-Nigeria. High satisfaction was expressed by a minority of 42% and factors such as income, length of employment and length of enrolment slightly influenced satisfaction. Marital status, general awareness of contributions and benefits significantly and positively influenced clients’ satisfaction.

#### NHIS (Ghana)

The main NHIS elements of the Ghanaian healthcare system are shown in shaded boxes of Figure 2. The NHIS can make use of three types of health insurance scheme: the District Mutual Health Insurance Schemes (DMHIS), the Private Mutual Health Insurance Schemes (PMHIS), and the Private Commercial Health Insurance Schemes (PCHIS). The Scheme’s objectives, as implemented centrally and locally by the National Health Insurance Authority (NHIA), are to ensure access to basic health care services to all residents of Ghana with the following objectives: (1) register, license and regulate health insurance schemes, (2) supervise the operations of health insurance schemes, (3) grant accreditation to healthcare providers, (4) monitor compliance, and (5) perform other functions conferred upon it under Act 650 and Regulations 1809. In 2010, there were 145 NHIA-accredited DMHISs and 574 accredited providers across the 10 States of Ghana (see Figure 2 for categories). The NHIA was also reviewing applications from the private health insurance schemes in 2012 [7]; at the time of publication some PMHISs are fully operational within the NHIS [39].

Starting with revenue-raising, it can be seen that the central plank of the system is the National Health Insurance Fund (NHIF), which pools funding revenues from a number of sources as follows:

•Government of Ghana (GoG) - derived from taxation (direct and indirect), which includes subsidies for those exempted from premiums

•2.5% National Health Insurance Levy (NHIL)

•2.5% Social Security and National Insurance Trust (SSNIT) as deductions at source from formal sector employees

Other funding includes returns from investment, DF (as shown in the arrow of Figure 2) and premiums collected at the State level for non-formal sector employees. Revenues from the NHIF are then used to provide a ‘reinsurance mechanism’ for the DMHISs [25] and premiums for exempt groups, which are: (1) SSNIT contributors, (2) SSNIT pensioners, (3) people aged 70 years and above, (4) children under 18 years, (5) indigents (those who are poor with no source of income or fixed abode). An ID card facilitates access to services after a specified waiting period.

Full time students over 18 years are required to pay the minimum informal sector premium to obtain benefits. Spouses of SSNIT contributors who are not themselves SSNIT contributors must also pay a premium set for the informal sector. In 2012, the informal sector premiums ranged from GHC7.2 to 48.0 (US$ 3.8-25.3; 2012 values) per annum and are intended to be determined by the governing board of each DMHIS. In theory this should mean that premiums in the informal sector are progressive but there have been difficulties in determining accurate income figures. Therefore, flat rates have been commonly applied, which has meant this element of revenue-raising has been shown to be regressive [20].

The husband and all the wives in polygamous households have to be covered by NHIS for their children to be registered but, unlike the limited number of children in the Nigerian system, there is no limit of children per couple in Ghana. The system is generous as members do not pay any co-payments or deductibles.

The pooling of funds affords some sharing of risks among members but there is no risk-equalisation mechanism between the individual DMHISs. The NHIS secretariat allocates funds to DMHISs based on the number of SSNIT registered members as well as indigent members that have registered. Taxes are centrally collected, pooled and allocated to regional and district levels using a needs-based resource allocation formula [1]. As such, the issue of vertical equity is at least partially dealt with in relation to pooling within government agencies that purchase health care in the ten States of Ghana.

As shown in Figure 2, the NHIS separates the purchasing and provision functions. The DMHISs reimburse providers on a fee-for-service principle, based on the Ghana Diagnostic Related Groupings (G-DRGs) and a drug tariff list [25]. Public and some not-for-profit private (e.g. Christian Health Association of Ghana) facilities are allocated budgets and staff are paid salaries. Private for-profit practitioners are paid on a fee-for-service basis, where applicable, through OOP payments [1].

In terms of provision, the NHIA mandates a pre-defined benefits package that covers ‘95% of the disease burden in Ghana’ [7]. Services must be obtained at accredited facilities, which utilise gate-keeping between primary and secondary/tertiary care. In contrast to the Nigerian NHIS, the basic benefit package is the same for all DMHISs and membership categories. Like Nigeria, however, there are similar exclusions, including: (1) cancers other than cervical and breast cancers, (2) dialysis for chronic renal failure, (3) services covered under government vertical programs (including immunization, family planning and antiretroviral drugs), (4) drugs not listed on the NHIS Drug list, and (5) HIV/AIDS.

Every DMHIS establishes contracts with accredited providers to deliver services to its members, and reimburses providers after submission of claims for services. As with the Nigerian NHIS, the NHIS of Ghana has some limitations with regard to vertical and horizontal equity in access due to the exclusions of cover for some conditions.

#### NHIS implementation and factors affecting equity

In rather stark contrast to Nigeria, NHIS membership in Ghana has been rising sharply since its implementation. In 2007 membership was 35% [8] and by June, 2010, 66.4% of the population were registered (15,555,816 people) and there were 5,000 accredited providers. While SSNIT members accounted for about 6.4% (a figure that looks similar to the predominantly formal sector NHIS membership in Nigeria) 29.6% were in the informal adult sector, 48.9% were under 18 year-olds, 6.5% were 70 years or more, 6.7% were expectant mothers and 2.3% were indigent poor [7]. These data clearly show a substantial level of success in expanding membership when compared with Nigeria. The eight studies included in the review for Ghana, as shown in Table 3, are outlined in the following.

### Equity in financing

Three studies specifically addressed the issue of equity in financing in Ghana. A study by Akazili *et al.*[20] is highly informative to both Ghana and Nigeria as it used empirical analyses of households and other sources, coupled with well-established methods to determine the degree of progressivity in different categories of revenue-raising. The overall finding was that financing is progressive because of the major role of taxes. The NHI levy was found to be mildly progressive as were formal sector NHI payroll deductions. Informal sector NHI contributions, however, were found to be regressive. In spite of the emergence of the NHIS, OOP payments (45% of total health expenditure), were confirmed as being regressive. At the time of the analysis, NHIS premiums only accounted for 5% of total health care funding. Among other recommendations, the authors called for measures to expand the informal sector, possibly through a greater reliance on taxation, and the prepayment pool needs to increase in order to enhance budgetary allocations.

A study by Nguyen *et al.*[8] found evidence of the financial protection effect of the NHIS in that payments for care and uncovered drugs and tests occurred in the NHIS, but at significantly lower levels than the uninsured. The effect was strong among the poorest in the sample. However, OOP payments were not eliminated by social insurance programmes. Another study by Witter and Garshong [30] confirmed the reliance of the NHIS on tax-based funding at 70-75% of total funding and that the exempted group is substantial at about 30%. There was also evidence, however, of a growth in distressed DMHISs as the NHIA has been required to make further reinsurance payments to meet gaps between revenues and claims. In contrast to the findings of Akazili (*ibid*) the VAT-based levy in this study was found to be regressive.

### Equity in access

The issue of equity in access to health care in Ghana was addressed by six studies. The pro-rich bias and need to locate the poor for exemption was explored in two studies by Aryeetey *et al.*[40,41] who evaluated the equity, efficiency and feasibility of different strategies. They found that the cost to the government of exempting one poor individual from premiums was quite high (US$15.87 to US$95.44) and that means testing (MT) of wealth and geographic targeting (GT) were the optimal mechanisms depending on the setting (poverty level and rural/urban characteristics). These studies highlight the need of similar countries that require identification and exemptions of the poor in SHI programmes if universal coverage is to be achieved.

Two studies by Jehu-Appiah *et al.*[25,42] involved surveys of over 3,000 households and their perceptions of the NHIS according to insured or uninsured status, and the demand for membership according to socio-economic status. The principal findings were that scheme factors such as price, convenience, provider attitudes, peer pressure and the benefits package are relevant in NHIS membership and retention. The uninsured were also found to be more negative than members with regard to these factors and there is evidence of inequity in enrolment in the NHIS according to socio-economic factors as clear differences exist between the rich and poor. A study by Sarpong *et al.*[43] also confirmed these findings among households in the Ashanti region of Ghana where, among the 38% enrolled in the NHIS 21% were low, 43% middle and 60% high socio-economic status households and that the association was significant. The work undertaken by Witter *et al.*[30] also provided supporting evidence that membership of the NHIS is pro-rich and pro-urban and that there is ‘squeezing out’ of non-members from access to health care.

In terms of improved outcomes through better access, a study by Mensah *et al.*[44] is of much interest as it evaluated the effect of the NHIS on MDGs 4 and 5 in women of child-bearing age in the administrative regions of Brong Ahafo and Upper East. Analysis of 400 NHIS members and 1,600 non-NHIS members showed that women enrolled in the NHIS were more likely to receive prenatal care, deliver at a hospital, have their deliveries attended by trained health professionals, and experience less birth complications. A study by Nguyen *et al.*[8] also identified the need for improvements in supply-side incentives and the quality of care.

## Discussion

We commenced this study by re-affirming standard definitions of equity in health care, and outlining the chosen analytical approach. The principal reason for choosing the WHO framework [12] was that it facilitates a means of diagrammatically representing how the NHIS of each country fits into other elements of the healthcare system with regard to the common functions of revenue-raising, pooling, purchasing and provision. These methods enabled us to capture the well-recognised complexity and fragmentation of health care in Nigeria and Ghana. Although the full analytical framework includes other common elements of *stewardship* and *resource generation*, which are vertically integrated throughout any healthcare system [12], these were not the focus of our attention although our findings have encompassed stewardship of each NHIS to a limited degree.

Our analysis of health and economic outcomes at a macro level over the period 2000–2010 has captured some informative trends pre- and post-NHIS implementation. Nigeria and Ghana are both making positive progress and have transitioned to LMIC status, with GDP growth rates well above those of most developed countries. This suggests a growing capacity to fund and deliver health care, which is confirmed by comparable per capita health care spending for both countries. However, as a share of GDP both countries spend less than half the OECD mean, which implies that there is room for greater increases in health spending that could drive equity improvements in health care.

Both countries have also steadily improved their health outcomes over the period examined but the findings reveal strong inequalities, with a sustained or growing advantage for Ghana. Using the raw data of Table 1 for 2000 and 2010, respectively, it can be determined that life expectancy in Ghana was 20.8% and 23.5% higher (males), and 25.5% and 25% higher (females); infant mortality was 44.8% lower and 43.2% lower in Ghana; under-5 year mortality was 47% lower and 48% lower in Ghana; the prevalence of HIV was 41% lower and 50% lower in Ghana; the incidence of TB was 11.6% lower and 35.3% lower in Ghana. However, improvements in health outcomes cannot be attributed solely to the efforts of the healthcare system as health production theory indicates that other factors influencing health outcomes include environment, lifestyle and human biology [11]. This is an important consideration as per capita spending on health in Nigeria has been very similar or higher over the period 2000–2010 (Table 1), which may also suggest greater efficiency in the Ghanaian healthcare system. In this respect, the MDGs of the United Nations are clearly important as they provide motivation to improve health indicators through targets that seek to eradicate the underlying causes of premature mortality, including poverty. MDGs now form an integral part of national development policy for Nigeria and Ghana as reflected in their regular MDG reports [18,19]. Differences in health attainment between Ghana and Nigeria, including greater inequalities across the Nigerian population, were confirmed in the WHO Report of 2000 [23].

The issue of equity in fund raising is clearly relevant regarding health outcomes as the findings of this study show it has an impact on access to health care. The findings for macro-level data reveal more beneficial equity developments in Ghana pre- and post-NHIS implementation; OOP health expenditure as a percentage of private health expenditure decreased by 14% in Ghana while in Nigeria there was an increase of 2%; public health expenditure as a share of total health expenditure increased in Ghana by 19% while in Nigeria there was a modest 5% increase. As a share of total expenditure the greater reliance on this regressive form of funding is confirmed by figures of 65% for Nigeria and 45% for Ghana. Removing such a strong reliance on OOP expenditure is key to improving equity in financing [1,20] - the WHO indicates this should less than 15–20% of total health expenditures in order to prevent financial catastrophe regarding access to health care [3], which both countries are clearly well short of.

The NHIS of Nigeria and Ghana explicitly seek to provide prepayment protection against the ill-effects of OOP payments by widening membership. In this regard we have observed a very substantial difference in success, with Ghana achieving 65% and Nigeria only 3.5% population coverage. In discussing this difference it is worth considering the challenges the Nigerian NHIS faces through its three-tier arrangements for the formal, urban self-employed and rural programmes. While the formal sector programme is (theoretically) statutory and easier to implement through payroll deductions, the other programmes are voluntary and broadly based on the CBHI model, which is to be scaled up with the advantages of minimum membership (to assure sufficient funding and risk sharing), regulated administration and NHIS-accredited providers. The general aim is to bring existing and new ‘user groups’ under the NHIS umbrella. Additional merits of this model include its not-for-profit basis and possibly increased social cohesion and success when groups with a common interest come together, but the known issues of low participation rates, a limited benefits package, adverse selection (of high risks) and the use of regressive contributions make its expansion potential problematic. The promised good management provided under the NHIS will therefore be essential, especially in remote regions with high rates of poverty. Widening membership through targeted exemptions for more sub-groups in Nigeria using general taxation, as is more prominent in Ghana, may be necessary if substantial moves towards universal health care are to be achieved.

The common feature of donor funding raises some potential conflicts of interest and adverse effects concerning equity because, while it may bolster access to health care at various levels of the system (Figures 1 and 2), it may also be transient and poorly targeted. According to the WHO, it also has the capacity to dampen the supply-side efforts of governments as they may be unwilling either to spend more on health or do not acknowledge that they have the capacity to expand prepayment and pooling systems [3]. Our findings also indicate that it is more generous towards Ghana at about three times higher than Nigeria. Interestingly, however, it has recently been announced that the International Finance Corporation (IFC) has signed a US$2.3 million agreement with the Nigerian government that is aimed at speeding up the development of health insurance. The Government’s aim is to fast-track the enrolment of the majority of Nigerians into the NHIS [45]. While this is encouraging in the short-term, increased health care expenditure from the Government must surely be a more sustainable means of widening membership.

The findings of the review for Nigeria uncovered mixed messages regarding the attributes of the NHIS but clearly confirmed the negative impacts of OOP expenditure, which limits or causes delays in access, and encourages the use of potentially ineffective alternatives or self-denial of treatment. NHIS membership exhibits a clear urban/rural divide in uptake and socio-economic factors are significant in terms of participation and therefore access. Encouragingly, however, there is a good level of willingness to participate in the NHIS, especially in rural areas where it is hindered by a lack of knowledge regarding the details and benefits of the NHIS. The evidence confirms that the Nigerian government needs to do more in terms of advertising and promoting the NHIS in remote parts of the country to expand membership. A major issue affecting equity in access is that the benefits packages for the three programmes that constitute the NHIS are not uniform and more comprehensive coverage is provided in the formal sector.

The review findings for Ghana reveal a number of advantages with respect to equity compared with Nigeria. In addition to its rapid increase in membership, equity in access is enhanced through clearly-defined exempted groups that are funded from general taxation. There are also financial protection effects and improved access to health care with some evidence of improved health outcomes. The issues that adversely affect equity include the need to identify and promote membership for the rural poor, dealing with the pro-urban and pro-rich membership, and the presence of distressed DHMISs, which may partly be the result of moral hazard as there are no (or minimal) co-payments to dampen demand to partly control costs [11]. Another advantage concerning provision for Ghana comes from the fact that the benefits package is the same for all members, which gives Ghana a clear advantage in equity regarding access and provision, but it also has a number of exemptions that adversely affect vertical equity in particular.

In spite of Ghana being seen as a ‘leading light’ in this study and more generally in the literature, a recent report from Oxfam has generated much debate as it questions the coverage and benefits claims regarding Ghana’s NHIS [46]. Early assessments of the NHIS in Ghana also found cases of NHIS card holders experiencing long delays for treatment, being required to make unofficial additional user charges, and receiving poorer quality care [8].

The results of our literature review revealed a number of informative additional studies of Sub-Saharan African countries with regard to equity in financing and initiatives to achieve universal health care. In assessing the success of the NHIS in Nigeria, for example, Nnamuchi [47] likens it to a ‘white elephant’ and argues that it lacks the requisite policy and legal frameworks for realising many of the goals it sets out to accomplish, and that re-calibration is necessary. Sambo and colleagues [48] report opinions among high level policy makers in Africa that support the findings of the present study as they confirm the predominance of OOP spending, underdeveloped prepaid health financing mechanisms, large informal sectors vis-a-vis small formal sectors, unpredictability and non-alignment of the majority of donor funds with national health priorities, and the need to allocate at least 15% of the national budget to health care.

The work of Akazil and colleagues [20] is deserving of particular acknowledgement as their comprehensive study of equity in financing in Ghana serves as a model for much-needed analyses of other Sub-Saharan African countries. The work of McIntyre and colleagues [1] has been particularly beneficial in the present study as it provides in-depth analyses of Ghana, South Africa and Tanzania, in seeking a path from ‘fragmentation to universal health care.’ The WHO also clearly acts as a principal driver of universal health care through its numerous reports and as a final overview we would echo its recommendations for countries such as Nigeria and Ghana to: (1) raise sufficient funds in an equitable, efficient and sustainable manner, (2) reduce financial barriers through affordable access and equitable and efficient pooling, and (3) use resources wisely through their equitable and efficient use [3].

We believe that our study offers originality due to the head-to-head comparison of two countries with differing approaches and levels of achievement, with Nigeria having perhaps the greatest potential to benefit. The application of the WHO framework to assess healthcare system performance is also a novel approach and we would argue a fruitful method to represent healthcare systems with respect to equity. We encourage its use in future studies of this nature in order to make analyses easier to assimilate for policy makers. In terms of limitations, whilst our review of the literature was based on a systematic search of electronic databases, websites and included studies, we have not explored the grey literature in detail. The two diagrams of Nigeria and Ghana presented in this study could also be developed further to improve their precision and comprehensiveness.

## Conclusions

Evidence from this study indicates healthy economic growth to LMIC status for both Nigeria and Ghana, with steady improvements in health indicators in recent years but there is a clear and sustained advantage for Ghana. Both countries have introduced NHISs with the aim of reducing the reliance on OOP payments and are looking for these systems to eventually provide universal health care for their citizens. Whilst progress is being made in both countries, the results strongly suggest that equity in relation to financing and access are weaker in Nigeria compared with Ghana. Nigeria particularly needs to take steps to expand membership outside the formal sector, possibly through exemptions using general taxation as has been more widely adopted in Ghana. Nigeria appears to have been receiving lower levels of donor funding but the latest arrangement with the IFC has offered greater expansion potential of NHIS membership. Nigeria clearly has the capacity to expand its own internal spending to achieve greater integration of its healthcare system through the NHIS. Heavy burdens of poverty, disease, remote rural settings and variability in provision across both Nigeria and Ghana present substantial challenges. Ghana’s success story is encouraging but has to be tempered by the high number of exemptions through taxation and the threat of moral hazard. Some recent evidence questions the level of success actually achieved. The WHO methods of healthcare system analysis, as applied in this study, are informative in this area of study and could be beneficially expanded in future analyses.

## Abbreviations

AIDS: Acquired immunodeficiency syndrome; DF: Donor Funding; DA: District Authority; CBHI: Community-based health insurance; CBO: Community-Based Organisations; CSO: Civil-Society Organisation; DALE: Disability-adjusted life expectancy; DMHIS: District Mutual Health Insurance Scheme; DRG: Diagnostic Related Group; FBO: Faith-Based Organisation; FSHIP: Formal Sector Social Health Insurance Programme; GoG: Government of Ghana; GDP: Gross Domestic Product; G-DRGs: Ghana Diagnostic Related Groupings; GT: Geographic targeting; HCP: Health Care Provider; HMA: Health Mutual Association; HIV: Human immunodeficiency virus infection; HMO: Health maintenance Organisation; ID: Identity (card); IFC: International Finance Corporation; LGA: Local Government Area; LMIC: Low and Middle-Income Country; MCH: Maternal and Child Health; MHIS: Mutual Health Insurance Scheme; MT: Means testing; NACA: National Action Committee for AIDS; NHIL: National Health Insurance Levy; NGO: Non-Governmental Organisations; MDG: Millennium Development Goal; NCH: National Council on Health; NHI: National Health Insurance; NHIA: National Health Insurance Authority; NHIF: National Health Insurance Fund; NHIS: National Health Insurance Scheme; OECD: Organisation for Economic Co-operation and Development; OOP: Out-of-pocket; PHI: Private health insurance; PCHIS: Private Commercial Health Insurance Schemes; PMHIS: Private Mutual Health Insurance Schemes; RCSHIP: Rural Community Social Health Insurance Programme; SHI: Social Health Insurance; SSNIT: Social Security and National Insurance Trust; TB: Tuberculosis; UK: United Kingdom; US: United States; VAT: Value-added tax; USSHIP: Urban Self-Employed Social Health Insurance Programme; WHO: World Health Organisation

## Competing interests

Isaac A.O. Odeyemi is an employee of Astellas Pharma UK Ltd. John Nixon has received consultancy and writing fees from Astellas Pharma Europe Ltd.

## Authors’ contributions

IAOO and JN contributed to the intellectual input, design, literature review, drafting and editing of the manuscript. JN initially constructed Figures 1 and 2, which were modified and updated after discussion with IAOO. Both co-authors read and approved the final manuscript.

## Authors’ information

JN is currently teaches an academic module for health care professionals at the university of York entitled the ‘Economics of Healthcare Systems.’
